# Clinical Features of Primary and Systemic Metastatic Intraocular Lymphomas in Spanish Patients

**DOI:** 10.1155/2019/6327041

**Published:** 2019-10-16

**Authors:** Victor Llorenç, Carla Fuster, Carmen Alba-Linero, Aina Moll-Udina, Alba Serrano, Manel Solé, Maite Sainz de la Maza, Iban Aldecoa, Alfredo Adán

**Affiliations:** ^1^Institute Clinic Ophthalmology, Hospital Clinic of Barcelona, Barcelona, Spain; ^2^Department of Pathology, Hospital Clinic of Barcelona, Barcelona, Spain

## Abstract

**Objectives:**

To describe and compare clinical findings in different subtypes of biopsy-proven intraocular lymphomas (IOLs).

**Design:**

Retrospective, observational case series.

**Methods:**

The clinical and pathologic features in IOLs at the Hospital Clinic of Barcelona from 1995 to 2018 were retrospectively studied.

**Results:**

Twenty-one patients, 12 men (57%), median age 60 (interquartile range, IQR: 18 years), and a median follow-up of 30 (IQR 60) months were included. Eleven patients had primary vitreo-retinal lymphoma (PVRL, 52%), 4 had primary uveal lymphoma (PUL, 19%), and 6 had systemic metastatic retinal lymphomas (SMRLs, 28%). Diffuse large B-cell lymphoma was the main IOL subset in PVRL (91%) and in SMRL (83%), whereas extranodal marginal zone lymphoma was the only type in PUL (100%). Survival rate was 44% in PVRL and 20% in SMRL at 5 years (*p*=0.047). One patient had flow cytometry of two different vitreous humour samples. With them, flow cytometry was performed in a total of 11 samples, yielding 7 positive samples.

**Conclusions and Relevance:**

Even though PVRL is the most frequent IOL subtype, our findings suggest that PUL and SMRL should be considered as potential IOL causes. Overall survival was poor in PVRL and even shorter in SMRL patients.

## 1. Introduction

Intraocular lymphomas (IOLs) are lymphoid system neoplasia arising from either within the eye (primary intraocular lymphoma, PIOL) or outside the eye, with subsequent intraocular infiltration (systemic metastatic retinal lymphoma, SMRL). Recommendations regarding nomenclature are primary or secondary intraocular involvement (systemic metastatic lymphoma) followed by initial intraocular affected tissue (primary vitreo-retinal lymphoma or PVRL and, primary uveal lymphoma or PUL). [1] However, the scientific literature that systematizes the different IOL subtypes remain scarce.

Intraocular lymphomas are rare malignancies. These are estimated to represent less than 1% of intraocular tumors and 1%-2% of extranodal lymphomas [2, 3]. An increase in primary central nervous system lymphomas and PVRL, as its subset, has been recorded in the last decades [2, 4]. Reasons for PVRL rise are unknown, but a longer life expectancy, increasing use of immunosuppressive therapies, and improvement in diagnostic techniques could be possible explanations.

Intraocular lymphomas are a major diagnostic challenge. The so-called classically “masquerade syndromes,” they use to present mimicking chronic uveitis in adult or elderly patients. Early diagnosis is crucial in IOLs because they can be life-threatening diseases with a completely different management than uveitis [3, 4]. Cytological diagnosis in ocular samples is still key for an early definitive diagnosis and challenging also from the pathologist perspective [5].

Herein we aim to describe and compare clinical and pathological features as well as survival rates in pathologically proven patients with IOLs.

## 2. Subjects and Methods

An observational, comparative study was conducted by retrospective chart-review of patients with IOLs at Hospital Clinic of Barcelona from 1995 to 2018. Inclusion criteria were patients with IOLs, either primary or systemic metastatic in whom a cytological/histological confirmation in ocular samples was obtained. A minimum follow-up of one year was required in survivors. All the methods adhered to the tenets of the Declaration of Helsinki. The study protocol was approved by our Institutional Review Board.

Patient's characteristics, ocular signs, visual acuity, therapeutic approaches, cytological and/or histological findings from ocular samples, and survival rates were recorded for analysis.

For intraocular samples retrieval, a 3-port pars plana vitrectomy was performed. Over 1 ml of direct undiluted vitreous was aspirated initially prior to start the infusion by connecting a 5-ml syringe. A second diluted vitreous sample was also collected during the vitrectomy procedure. Subretinal aspirate or retinal biopsies were also obtained in some cases when vitreous samples resulted negative and progressing retinal lesions appeared. Standard anterior chamber paracentesis by fine-needle aspiration was performed as a first intention when there was anterior intense cell reaction or pseudohypopyon. If a manifest iris or teno-conjunctival infiltration was detected, a biopsy of the most accessible suspicious involved tissues was done as a first diagnostic approach. Fresh unfixed samples were sent to the Pathology Department within 1 hour after personal advice to pathologist for rapid processing. Undiluted vitreous samples containing more than 1 ml were divided for cytological evaluation and flow cytometry. Diluted vitreous samples were centrifuged to obtain a pellet that, in turn, was divided for cytology and cell-block procedures. Samples for cytology were processed using Cytospin in or ThinPrep according to the manufacturer instructions. Slides were stained with Papanicolaou and Romanowsky based quick stains. Biopsy specimens were fixed in 4% formalin and were included in paraffin. Four micrometer sections were mounted on slides and stained with hematoxylin (Harris Hematoxylin, PanreacApplichem, ITW Reagents) and eosin (Alcoholic eosin 1% QCA). Immunohistochemical studies were performed during the initial diagnostic workup, and stains were selected based on diagnostic suspicion and sample availability. The antibodies performed were CD20, CD19, CD79a, CD2, CD3, CD4, CD5, CD8, CD10, Ki67, BCL2, and BCL6 using the manufacturers protocol (Dako, Glostrup, Denmark). Epstein–Barr encoding region (EBER) in situ hybridization (Roche Diagnostics, Indianapolis, USA, or Dako, Glostrup, Denmark) was also performed when needed. For flow cytometry, cells in suspension from vitreous humor specimens were incubated with combinations of surface antigens that included B-cell, T-cell, and NK-cell markers. Stained samples were acquired in a FACSCanto II using the FACSDiva software program (Becton Dickinson Bioscience, San Jose, CA), and data were analyzed with the Infinicyt software (Cytognos SL, Salamanca, Spain). From each specimen, the percentage of CD3+, CD5+, CD7+, CD4+, CD8+, CD57+, B-cells, and kappa/lambda ratio were calculated. In addition, when kappa/lambda ratio was abnormal, the coexpression of CD5 and CD10 in B-cells was studied. The diagnosis was based on the cytological morphology with the aid of immunohistochemistry and flow cytometry when available. To further evaluate the characteristics of the cytological samples, these were reassessed by three of the authors (MS, IA, and CF), using the morphologic criteria, as Rodriguez et al. described [6]. For comparisons among groups, nonparametric Kruskal–Wallis test and post hoc Mann–Whitney *U* test was applied for quantitative continuous and ordinal variables, and Fisher exact test was used to compare categorical data. To compare paired visual acuity proportions, the McNemar test was applied. Statistical significance was set at *p* < 0.05. Statistical analysis was performed with MedCalc® software, version 17.9.7 (MedCalc® bvba. Ostend, Belgium).

## 3. Results

From 25 identified patients in our institutional database, 4 had to be excluded because of missing data or loss of follow-up. Twenty-one patients (32 eyes) with an overall median follow-up of 30 (IQR 60) months were finally included for analysis. Fifteen cases (15/21, 71%) were classified as PIOL and 6 patients (6/21, 28%) as SMRL. In those patients with PIOL, two subgroups could be clearly differentiated: those infiltrating the vitreo-retinal tissues, or primary vitreo-retinal lymphoma (PVRL, *n* = 11) and those patients with primary uveal infiltration or primary uveal lymphoma (PUL, *n* = 4). There were not significant differences in follow-up time between groups (*p*=0.689 by Kruskal–Wallis test).

Patients were 12 men (12/21, 57%) and 9 women, with a median age at diagnosis of 60 (IQR 18) years old. We did not find any statistically significant difference regarding age and gender distribution. All patients were HIV negative. Most patients (18/21, 85%) were Caucasian, although patients with SRML were less often Caucasians than in the other groups (*p*=0.029). Eleven patients presented with bilateral IOLs (11/21, 52%). The disease was more frequently unilateral in cases of PUL (*p*=0.047). Median overall time from first ocular symptoms to ocular diagnosis was 2 (IQR 7.2) months. Patients with PUL showed longer time to diagnose than other subgroups (*p*=0.04) (Table 1).

### 3.1. Pathological Diagnosis (Table 2)

Twenty-five samples from 19 patients were received in the Pathology Department. The remaining two patients had a confirmation diagnosis in other centers. Four patients had two samples and one patient three. Eighteen specimens from 14 patients were analyzed for cytology: 15 vitreous samples, two aqueous, and one subretinal aspirate. Among those samples, 13 samples (13/18, 72%) were consistent with lymphoma, 3 were suspicious, and the other 2 were regarded as negative or reactive. In initially negative patients, a second positive vitreous cytology was found in one and a positive subretinal aspirate in the second patient. The most valuable cytological feature was the cell size, as nearly all the specimens had medium to large cells and, at least, mild nuclear irregularity. Pleomorphism was noted in 50% of the specimens, and to a lesser extent, the presence of apoptosis was noted. Mitotic figures, lymphoglandular bodies and presence of necrosis were not conspicuous features. Finally, accompanying inflammatory cells were frequent and present in at least low quantities in all but two (22/25, 88%) assessable samples. In the cytology specimens, most cases had ancillary studies, mainly immunohistochemical. Flow cytometry studies were performed in 11 samples of 10 cases. A sample was considered consistent with lymphoma when features such as restriction of Ig light chains or aberrant morphology with compatible immunophenotype were present, and suspicious with lymphoproliferative disorder when they were present but in scarce cells or was difficult to ascertain. In 7 samples, the rendered results were consistent with or suspicious for a lymphoproliferative disorder. A single patient had two vitreous humour samples (see Table 2); the one negative for cytology had a negative flow cytometry, while the one with SMRL DLBCL had a contributory flow cytometry. In the remaining 9 cases, 6 (or 67%) had a contributory flow cytometry (FC) (see Table 2).

In addition, seven biopsies were obtained: 3 teno-conjunctival, all of them with diagnosis of extranodal marginal zone lymphoma (ENMZL), also named as mucosa-associated lymphoid tissue B-cell lymphoma (MALT); one from iris tissue, found as a low-grade B-cell lymphoma (suggestive also of ENMZL); two from the retina and one enucleation, all three confirmatory of DLBCL. Diagnosis was supported by immunohistochemical studies in all the biopsies.

Final pathological diagnosis was DLBCL in 15/21, 71% patients (10/11, 91% PVRL and 5/6, 83% SMRL), T-cell lymphoma in one PVRL, netural killer T-cell lymphoma (NKTCL) in one SMRL and ENMZL in 4/4, 100% PUL cases.

### 3.2. Ocular Findings

**Figure 1:**
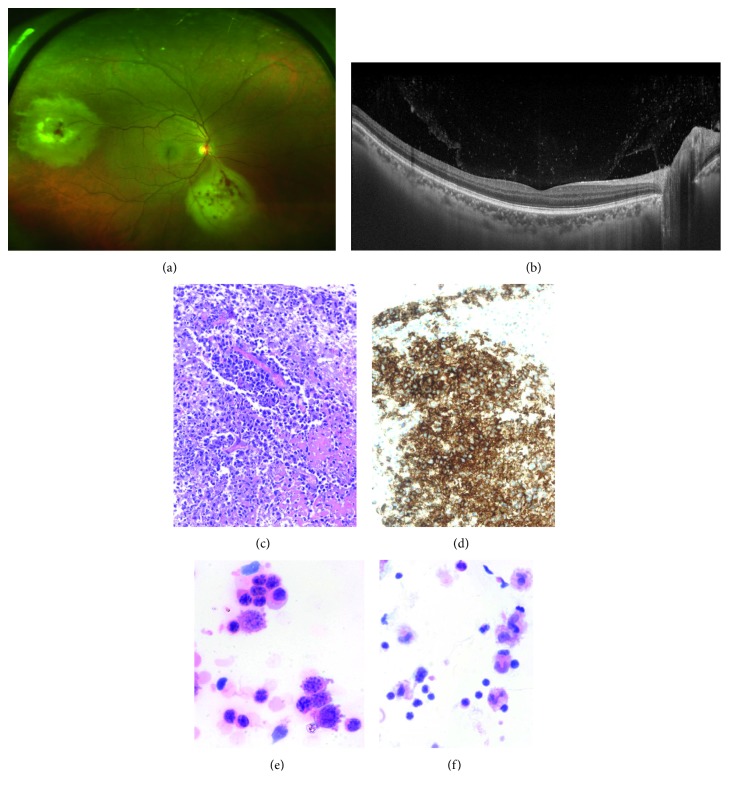
(a) Ultra-wide field pseudocolor retinography of the right eye of a 42 year-old black male. Currently in systemic remission, he was diagnosed with peripheral blood diffuse large B-cell lymphoma (DLBCL) 10 months before. Creamy whitish subretinal infiltrates with some hemorrhages suggested secondary metastatic retinal lymphoma (SRML). (b) Swept-source optical coherence tomography showed a sheet of vitreous cells. (c) Retinal biopsy (Hematoxylin-Eosin, 200x) confirmed intraocular diffuse large B-cell lymphoma. Note the large atypical cells admixed with coagulative necrosis. Bar size = 100 *μ*m. (d) The cell showed a diffuse and strong positivity for CD20. Bar size = 100 *μ*m. (e) Vitreous cytology (Diff-quick, 600x), atypical large cells with variable morphology and accompanying lymphocytes. Bar size = 50 *μ*m. (f) Vitreous cytology of the same sample (Diff-quick, 400x) showing multiple lymphocytes and macrophages. Bar size = 50 *μ*m.

**Figure 2:**
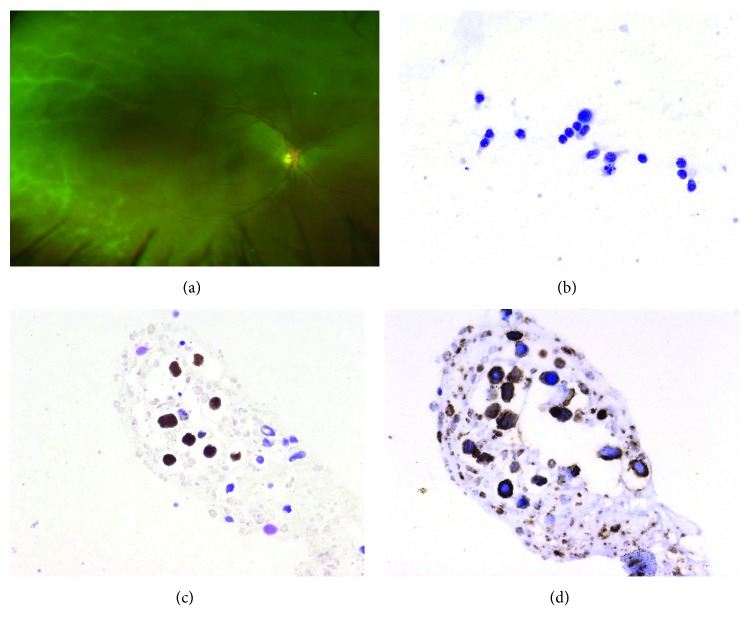
(a) Ultra-wide field pseudocolor retinography. Frosted-branch angiitis in the right eye of a 44-year-old woman complaining of blurry vision the last month. She has a completely remission of natural killer T-cell cavum lymphoma. (b) Vitreous cytology (Papanicolau 400x) confirmed atypical lymphoid cells. (c) In situ hibridation for Epstein-Barr Virus (EBER) was positive in the atypical cells (400x). (d) The cells showed a diffuse and strong positivity for CD3.

**Figure 3:**
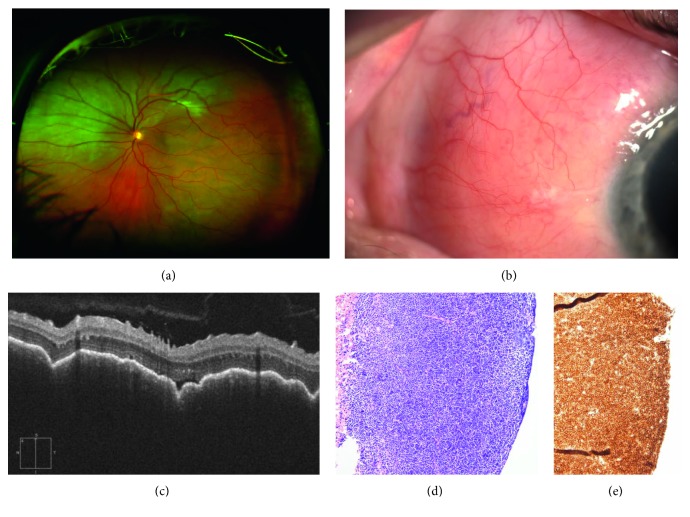
(a) Ultra-wide field pseudocolor retinography. Choroidal mass infiltration in the left eye of a 46-year-old man complaining of visual impairment during the past 36 months. (b) Anterior segment biomicroscopy showed subconjunctival salmon plaque, a primary uveal lymphoma with extraocular infiltration was suspected. (c) SD-OCT showed dome shaped pattern due to massive choroidal infiltration. (d) Teno-conjunctival biopsy (Hematoxylin& Eosin, 100x) yielded an extranodal marginal zone lymphoma, composed of sheets of homogeneous medium to small lymphocytes. Bar size = 100 *μ*m. (e) CD20 B-cell marker was strongly positive (100x). Bar size = 100 *μ*m.

Anterior chamber cells were seen in 15/32, 46% of the eyes, 2 of them with pseudohypopyon (one PVRL and one SMRL). Vitreous haze was found in 24/32, 75%, retinal involvement appeared in 9/32, 28% of the eyes, 7 of them with vasculitis-like lesions (7/32, 21%) and subretinal infiltration in 8/32 (25%) (Figure 1). Frosted-branch angiitis was observed in one Natural Killer T-cell lymphoma case (Figure 2). There were no significant differences between groups regarding anterior chamber cells, vitreous haze nor retinal/vascular involvement. In SMRL patients, median time from systemic lymphoma diagnosis to ocular involvement was 15 (IQR 52) months. All SMRL patients were considered to be in systemic remission before ocular diagnosis. Deep choroidal infiltration was seen in 4/4, 100% of the eyes with PUL, whereas only in 1/17, 5% of the eyes with PVRL (*p* < 0.001) and 1/11, 9% with SMRL (*p*=0.003) (Figure 3). Detailed ocular signs are described in Table 3.

Eyes with available best corrected visual acuity equal or worse than 20/200 (Snellen) increased from 5/27 (18%) of the affected eyes at presentation to 15/27 (55%) at the final follow-up. The worst visual outcome was noted in SMRL eyes, in which visual acuity ≤20/200 increased from 1/9, 11% of the affected eyes at presentation to 6/11 (66%) at the final follow-up. However, paired visual acuities did not show statistically significant differences in any group.

### 3.3. Extraocular Findings

Four patients with PVRL (4/11, 36%) presented with simultaneous subclinical CNS involvement at diagnosis and 3/11 (27%) patients with PVRL developed CNS disease during follow-up at a median of 6.5 (IQR 7) months. In three out of four (75%) patients with PUL, an undiagnosed subconjunctival salmon infiltrative plaque was discovered at the same time as intraocular uveal involvement. In SMRL patients, primary origin was lymph nodes in 3 (50%) cases and peripheral blood, pyriform sinus, and cavum in one case each. Four out of six (66%) patients with SMRL developed also extraocular metastasis either after intraocular involvement or simultaneously. Extraocular spreading in SMRL involved the CNS in 2 cases, CNS and bone-marrow in one, and lymph nodes in one.

### 3.4. Therapeutic Management

All patients in our study received systemic chemotherapy, mostly CHOP (cyclophosphamide-doxorubicin-vincristine-prednisone) (6/21, 28%), sometimes in combination with rituximab (R-CHOP) (6/21, 28%) as first line approaches. BRAM (Carmustine-rituximab-araC-methotrexate) schedule (3/21, 14%), rituximab (2/21, 9%), or high-dose methotrexate alone (2/21, 9%) were also applied as first line chemotherapies. In addition, intrathecal methotrexate for CNS prophylaxis was used in 7/21 (33%) of the patients. Furthermore, 4/21 (15%) of the patients received intraocular chemotherapy; one with rituximab, one with methotrexate, and two with both consecutively. Six patients (6/21, 28%) received also external ocular radiotherapy and six (6/21, 28%) reduced-dose whole-brain prophylactic radiotherapy. Nevertheless, more than half of the patients (12/21, 57%) were also treated with other rescue chemotherapeutic schedules for ocular and/or extraocular relapses. Statistical differences regarding treatment could not be studied due to very different approaches among patients, eyes, and groups.

### 3.5. Patient's Survival

At the final follow-up, 12/21 (57%) of the patients died. Death causes were CNS involvement in 9/12 (75%) holocraneal radiotherapy in one, pneumonia in one, and unknown cause in the last patient. Survival rates were worse in the SMRL group and resulted significantly worse at the final follow-up (*p*=0.047) and at 5 years (*p*=0.047) (Table 1).

## 4. Discussion

Only a few published series describe and compare different subtypes of IOLs. Among IOLs, PVRL is the most described type of IOL by far, accounting for 72% as recently described [7] and 52% in our study. PUL are described rarely and usually excluded in IOL series, or reported separately [4, 8]. Nevertheless, PUL were found in 19% of our patients. Intraocular relapse of a systemic or secondary lymphoma has also been described as anecdotal; however, they are not so in our experience, conferring a poorer vital prognosis since they represented 28% of our IOL cases, as much as Karakawa et al. reported [7, 9].

In PVRL patients, we found a median age at presentation of 60 (IQR 12.5) years with a slight female preponderance (6/11, 55%). All patients were Caucasian. Higher incidence in women have been described by Cassoux et al. [10]. Nevertheless, demographical data in our study matched very well with previously published PVRL series [4, 5, 11]. As classically described, main ocular signs found in our PVRL patients were vitritis (76%), retinal (29%), and/or subretinal (29%) yellowish infiltrates [12]; anterior chamber cells greater than 1+ (SUN scale) was only seen in one eye. We also found one eye with hemorrhagic pseudohypopyon, and 17% of the eyes showed some degree of retinal vascular involvement. Pseudohypopyon has been rarely found previously in PVRL and retinal vasculitis is a well-known sign found in some cases [13, 14]. Central nervous system involvement was discovered during the extension exams in 36% of the patients with PVRL whereas 27% of the patients developed brain lesions at a median of 6.5 (IQR 7) months. CNS involvement has been found in 42% to 92% within a mean interval of 8–29 months [4, 11].

In our study, 64% of the flow cytometry studies rendered results consistent with or suspicious for a lymphoproliferative disorder. Cantu et al. [15] observed that the absence of large lymphocytes frequently demonstrates negative flow cytometry immunophenotyping, being the sole cytologic feature significantly associated with a negative result.

PVRL are usually B-cell phenotype and fall within the category of DLBCL; isolated PVRL of T-cell origin occur rarely, and poorer prognosis than in DLBCL has been suggested [3, 16]. We found one case with T-cell PVRL. The patient developed CNS lesions 3 months after ocular diagnosis with low response to systemic high-dose methotrexate-based chemotherapy and reduced-dose whole-brain radiotherapy and died 9 months later.

Survival in PVRL is poor and variable depending on follow-up time and case series, ranging from 91% to 19% at 12 to 35 months [4]. Grimm et al. [17] found an overall survival of 31 months in 221 patients with PVRL and CNS involvement with different treatment regimens, Lee et al. [18] found a survival of 19.7 months, and Cho et al. [19] an overall survival of 31 months, but it decreased to 18 months in concurrent PVRL/CNS involvement. They concluded that intraocular chemotherapy had not impact on survival [17, 18]. We were not able to compare treatment schedules due to its variability between patients and between eyes from the same patient, but overall PVRL survival was 24 (IQR 59.5) months at 25 (IQR 59.5) months of follow-up, not very different than previously reported overall survival time [20, 21]. Recently, Kaburaki et al. [22] suggested an improvement in overall survival (86.3% at 4 years) with combined intravitreal methotrexate and immunochemotherapeutic followed by reduced-dose whole-brain radiotherapy in B-cell PVRL with and without CNS involvement. However, overall survival was only slightly worse in patients with PVRL in our series, dropping down from 81% at 1 year to 66.6% at 4 years, and 44% at 5 years, despite different therapeutic approaches were used in those eyes/patients.

Primary uveal lymphoma was diagnosed in 4 men around their 6^th^ decade, all Caucasian. Disease was unilateral in all of them and diagnosis was delayed a median of 9 (IQR 20.5) months, significantly longer than in other types of IOL. PUL course was slowly indolent and undiagnosed teno-conjunctival infiltration was found in 3/4 (75%) of them, leading to easy biopsy and diagnosis. An iris biopsy was necessary in the last patient, demonstrating a B-cell ENMZL in all of them. Response to external ocular radiotherapy or systemic rituximab was excellent with 100% survival at 5 years, disease remission and preserved visual acuity in all cases. Good long-term prognosis has been described in PUL, although it can lead to permanent blindness without early diagnosis and proper treatment [23, 24]. Aside of characteristic deep uveal infiltration, anterior segment should be carefully explored in these eyes, because they can provide accessible samples for definitive diagnosis [25–27].

Secondary intraocular lymphomas were seen in both sexes around their 4^th^-5^th^ decade. They were seen in non-Caucasian patients more often than in other IOL subtypes and there was a trend towards an earlier diagnosis with a median of 1 (IQR 0.5) month. A previous history of systemic lymphoma could be the clue for early clinical suspicion. Vitreo-retinal bilateral involvement was the most frequent presentation of SRML, in line with Salomao et al. findings [28]. There was a trend towards a higher anterior chamber cell and vitreous haze grades with granulomatous keratic precipitates in SRML, as suggested previously by other authors. [3] All but one SMRL case fell into DLBCL type, with lymph node as a primary origin in half of them. Testicular origin was not detected in any case in our series, unlike it was previously described in 25% of SMRL cases [7]. Intensive rescue chemotherapies failed in most patients with SMRL, they were often bilateral (83%), and relapses were constant either intraocular or systemic [28]. Thus, survival was significantly poorer than in other IOL types in our series, 13.5 (IQR 20) months at 23 (IQR 36) months of follow-up. Intraocular relapse of a systemic lymphoma should be interpreted as an ominous sign of progression in our experience.

Fortunately, survival rates are increasing nowadays [29]. Cytological analysis of the vitreous was the most efficient diagnostic procedure in our series. Since most cases of IOL are DLBCL, diagnostic by simple morphological observation is straightforward provided that the specimen is adequately processed [30]. Nevertheless, although some authors do not support to do immunohistochemical studies in all cases [15], we support that immunohistochemical studies are mandatory and particularly important in more infrequent subtypes, such as NK/T‐cell lymphoma [31]. Other ancillary techniques can be performed when enough sample can be allocated to them. PCR techniques to detect clonality in either B-cell or T-cell lymphomas have been widely described in the literature [32]. The detection of gene rearrangements of IgH in B-cell lymphomas and TCR in T-cell lymphomas have been described in intraocular samples [21, 33], but the diagnostic yield of the technique can be very limited in intraocular samples, as they tend to be samples with scarce neoplastic cellularity with abundant accompanying cells, such as reactive lymphocytes, leucocytes, and macrophages [6]. Of note, we performed IgH rearrangement in one retinal biopsy of a DLBCL with secondary ocular involvement, and in a uveal biopsy of a low grade B-cell lymphoma but failed to detect a neoplastic clone in any of them. Nevertheless, the combined evaluation of cytological features and immunohistochemical phenotype enabled us to render confirmatory or suspicion diagnoses among the whole cohort of cases. Flow cytometry in cytological samples is a feasible procedure and theoretically provides more information, since it allows to study a wider range of diagnostic markers. Being a retrospective study, and although we try to perform it in every sample with suspicion of lymphoproliferative disorder, we could perform flow cytometry in only 11 samples of 10 patients. It gave supportive data in almost two thirds of the samples of our series. Of note, all the flow cytometry suspicious or positive case already had a positive pathological diagnosis. Hence, flow cytometry did not harbour higher sensibility, in line with other authors that have found a marked relationship between positive cytology and contributory flow cytometry results [15]. Biopsy samples enabled to confirm the clinical suspicion of lymphoma, but they are limited to safely accessible tissues or high clinical suspicion with previous inconclusive cytological findings.

Follow-up time, even though longer than other series, retrospective design, and relative low number of cases are the main limitations of this study.

In conclusion, PVRL are the most frequent IOL subtype (52%), although PUL and SRML are not as anecdotal as previously described, accounting for 19% and 28% of IOL in our series, respectively. DLBCL are the leading cytopathological subset in PVRL (91%) and SMRL (83%) whereas PUL is ENMZL in nature. Overall survival rates were low in PVRL (36% at 25 IQR 59.5 months), mainly due to CNS involvement (63% of patients), and significantly lower in SMRL (16% at 23 IQR 36 months) (*p*=0.047). An international multidisciplinary collaborative group on IOL management would be desirable to study treatment options and improve survival rates in this unusual disease.

## Figures and Tables

**Table 1:** Demographics, timing, and survival in patients with primary and systemic metastatic retinal lymphomas.

Subtype *n* (patients) (%)	PIOL *n* = 15		SMRL *n* = 6	Total *n* = 21
	PVRL *n* = 11	PUL *n* = 4		
Age, years	60 (12)	58 (26)	49.5 (17)	60 (18)
Gender (% males)	5 (45)	4 (100)	3 (50)	12 (57)
Caucasian, *n* (%)	11 (100)	4 (100)	3 (50)	18 (85)
Bilateral, *n* (%)	6 (54)	0 (0)	5 (83)	11 (52)
Follow-up, months	25 (59)	66 (12)	23 (36)	30 (60)
Survival, months	24 (59)	60 (6)	13.5 (20)	24 (58)
Survival at final follow-up	4 (36)	4 (100)	1 (16)	9 (42)
Survival at 1 year	9 (81)	4 (100)	4 (66)	17 (80)
Survival at 5 years	4/9 (44)	4/4 (100)	1/5 (20)	9/18 (50)
Time to ocular diagnosis, months	3 (7)	9 (20)	1 (0.5)	2 (7.2)

PVRL, primary vitreo-retinal lymphoma; PIOL, primary intraocular lymphoma; SMRL, systemic metastatic retinal lymphoma; PUL, primary uveal lymphoma.

**Table 2:** Contribution of diagnostic techniques according to final diagnosis and type of specimen in intraocular lymphomas.

Patient	Final diagnosis	Sample	Pathological diagnosis	IHC contributory	FC contributory
1	PIOL T	Vitreous	Suspicious	No	NP
2	PIOL DLBCL	Vitreous	Lymphoma	NP	NP
3	PIOL DLBCL	Vitreous	Lymphoma	Yes	No
3	PIOL DLBCL	Vitreous	Lymphoma	Yes	NP
4	PIOL DLBCL	Aqueous	Lymphoma	Yes	Yes
5	PIOL DLBCL	Vitreous	Lymphoma	Yes	NP
6	PIOL DLBCL	Vitreous	Lymphoma	Yes	NP
7	PIOL DLBCL	Enucleation	Lymphoma	Yes	NP
8	PIOL DLBCL	Vitreous	Lymphoma	Yes	NP
8	PIOL DLBCL	Vitreous	Lymphoma	Yes	Yes
9	PIOL DLBCL	Vitreous	Lymphoma	Yes	Yes
10	SIOL T-NK	Vitreous	Lymphoma	Yes	Yes
11	SIOL DLBCL	Vitreous	Negative	NP	NP
11	SIOL DLBCL	Subretinal aspirate	Lymphoma	Yes	NP
11	SIOL DLBCL	Retinal biopsy	Lymphoma	Yes	NP
12	SIOL DLBCL	Aqueous	Suspicious	Yes	NP
13	SIOL DLBCL	Vitreous	Negative	NP	No
13	SIOL DLBCL	Vitreous	Lymphoma	Yes	Yes
14	SIOL DLBCL	Vitreous	Lymphoma	Yes	Yes
15	SIOL DLBCL	Vitreous	Suspicious	No	No
15	SIOL DLBCL	Retinal biopsy	Lymphoma	Yes	NP
16	PIOL MALT B	Teno-Conjuntival biopsy	Lymphoma	Yes	NP
17	PIOL MALT B	Teno-Conjuntival biopsy	Lymphoma	Yes	No
18	PIOL MALT B	Iris biopsy	Lymphoma	Yes	Yes
19	PIOL MALT B	Teno-Conjuntival biopsy	Lymphoma	Yes	NP

PIOL, primary intraocular lymphoma; SIOL, secondary intraocular lymphoma; DLBCL, diffuse large B-cell lymphoma; NK, natural killer; MALT B, mucosa-associated lymphoid tissue B-cell lymphoma; IHC, inmunohistochemistry; FC, flow cytometry; NP, not performed.

**Table 3:** Ocular signs in primary and systemic metastatic lymphomas at presentation.

Subtype *n* (eyes) (%)	PVRL *n* = 17	SMRL *n* = 11	PUL *n* = 4
Anterior chamber cells^a^	7 (41)	6 (54)	1 (25)
>1+ (SUN)	1 (5)	2 (18)	0 (0)
Pseudohypopion	1 (5)	1 (9)	0 (0)
Fine KPs	1 (5)	1 (9)	0 (0)
Granulomatous KPs	0 (0)	2 (18)	1 (25)
Vitreous haze^b^	12 (70)	9 (81)	2 (50)
>1+ (NEI)	8 (47)	5 (45)	1 (25)
Retinal lesions	9 (52)	8 (72)	1 (25)
Vasculitis	3 (17)	4 (36)	0 (0)
Subretinal infiltration	5 (29)	3 (27)	0 (0)
Choroidal infiltration	1 (5)	1 (9)	4 (100)^*∗*^
Optic disk swelling	2 (11)	3 (27)	1 (25)

PVRL, primary vitreo-retinal lymphoma; SMRL, systemic metastatic retinal lymphoma; PUL, primary uveal lymphoma; SUN, Standardization of Uveitis Nomenclature scale; KPs, keratic precipitates; NEI, National Eye Institute scale. ^a^At any degree, ranging from 0.5+ to 3+. ^b^At any degree, ranging from 0.5+ to 4+. ^*∗*^*p* < 0.05.

## Data Availability

No data were used to support this study.
